# A massive immature mediastinal teratoma treated with chemotherapy and surgical resection: a case report

**DOI:** 10.1186/s13019-023-02389-w

**Published:** 2023-10-16

**Authors:** Parviz Mardani, Hooman Kamran, Rezvan Ghaderpanah, Bita Geramizadeh, Damoun Fouladi, Reza Shahriarirad, Armin Amirian

**Affiliations:** 1 Thoracic and Vascular Surgery Research Center, Shiraz University of Medical Science, Shiraz, Iran; 2 Students Research Committee, School of Medicine, Shiraz University of Medical Sciences, Shiraz, Iran; 3 Shiraz Transplant Research Center (STRC), Shiraz University of Medical Sciences, Shiraz, Iran; 4 Department of Pathology, Shiraz University of Medical Sciences, Shiraz, Iran

**Keywords:** Teratoma, Thoracotomy, Surgery, Chemotherapy, Neoplasms, Germ Cell and Embryonal, Mediastinal Neoplasms, Dyspnea, Chest Pain, Case Report

## Abstract

**Background:**

Teratoma is a type of germ cell tumor consisting of one or multiple tissues derived from germinal layers. The location and size of the tumor can cause various presentations. Here we report one of the largest ever cases of immature cystic teratoma.

**Case Presentation:**

In this report, we presented a 24-year-old patient with dyspnea, chest pain, nausea, and anorexia. A computed tomography scan revealed a giant, right-sided mass measuring about 190 × 150 × 140 mm. Chemotherapy was initiated for the patient, followed by thoracotomy. Histopathological evaluation revealed the nature of the mass to be an immature mediastinal teratoma.

**Conclusion:**

the incidence of immature mediastinal teratoma is uncommon, and due to its rarity, the diagnosis needs more profound evaluation studies such as radiological and pathological assessments. Immature teratomas are optimally treated by a combination of chemotherapy and complete resection.

## Background

Teratoma is a type of germ cell tumor consisting of one or multiple tissues derived from cells of three germinal layers, which may contain variable amounts of mature and immature tissues. Teratomas are mostly benign and are predominantly found in gonads; however, they can be identified in different anatomical locations such as the neck, retroperitoneum, sacrococcygeal region, and the thorax [1, 2].

Extragonadal germ cell tumors are most commonly located in the mediastinum and account for 15% of all mediastinal tumors in adults [3, 4]. The majority of the mediastinal teratomas are mature and develop slowly [5]. These tumors are usually asymptomatic at first, but as they grow in size, the patient may develop nonspecific symptoms like dyspnea and chest pain [6]. In this study, we report a 24-year-old male who presented with a massive immature mediastinal teratoma in the right lower lobe of the lung, which was successfully treated after lobectomy. To the best of our knowledge, our case is among the largest cases of immature cystic teratomas to ever be reported.

### Case presentation

A 24-year-old male was referred to our hospital complaining of a ten-month history of dyspnea, nausea, and anorexia, followed by a mild intermittent chest pain radiating to his back. He was a lifetime nonsmoker and denied any recent fever, chills, night sweats, or weight loss during this time. In physical examination, the patient was afebrile with normal vital signs, and oxygen saturation of 97%.

In his past medical history, first, the patient was visited regarding his signs and symptoms by a local physician, and high-resolution computed tomography (HRCT) scan was requested. The HRCT showed a large soft tissue mass in the anterior mediastinum with areas of coarse calcification (130 × 139 × 176 mm), causing the shift of heart and trachea to the left side and several lung nodules in both lungs up to 9.5 × 11 mm in the upper part of the lower lobe of the left lung, which could be metastatic lung nodules. The possible diagnosis was reported malignant teratoma and less likely other germ cell tumors. Besides, mild to moderate pericardial effusion was seen. He was referred to another city for further evaluation.

**Fig. 1:**
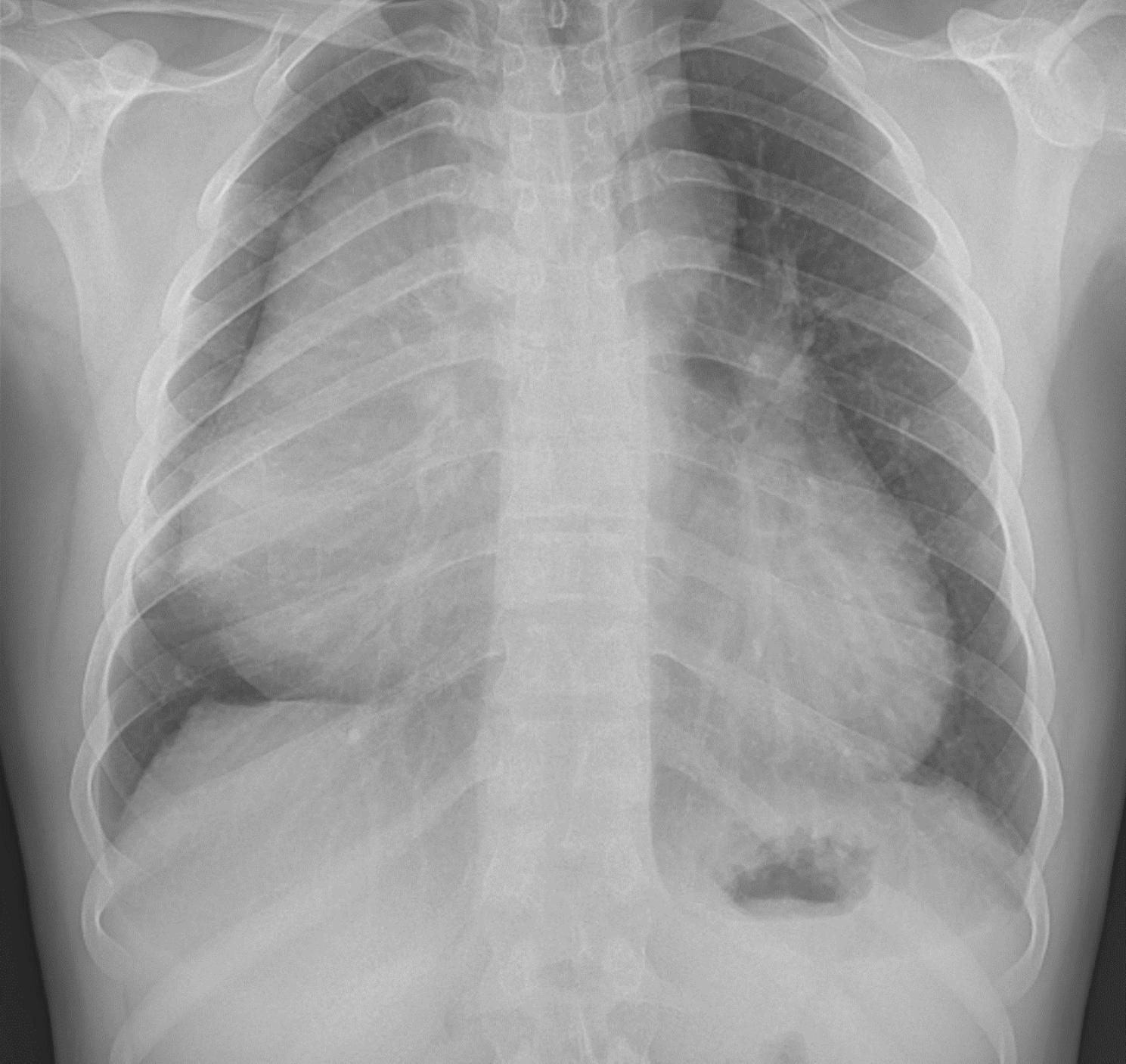
Chest x-ray showing a huge mass in the right mediastinum

**Fig. 2:**
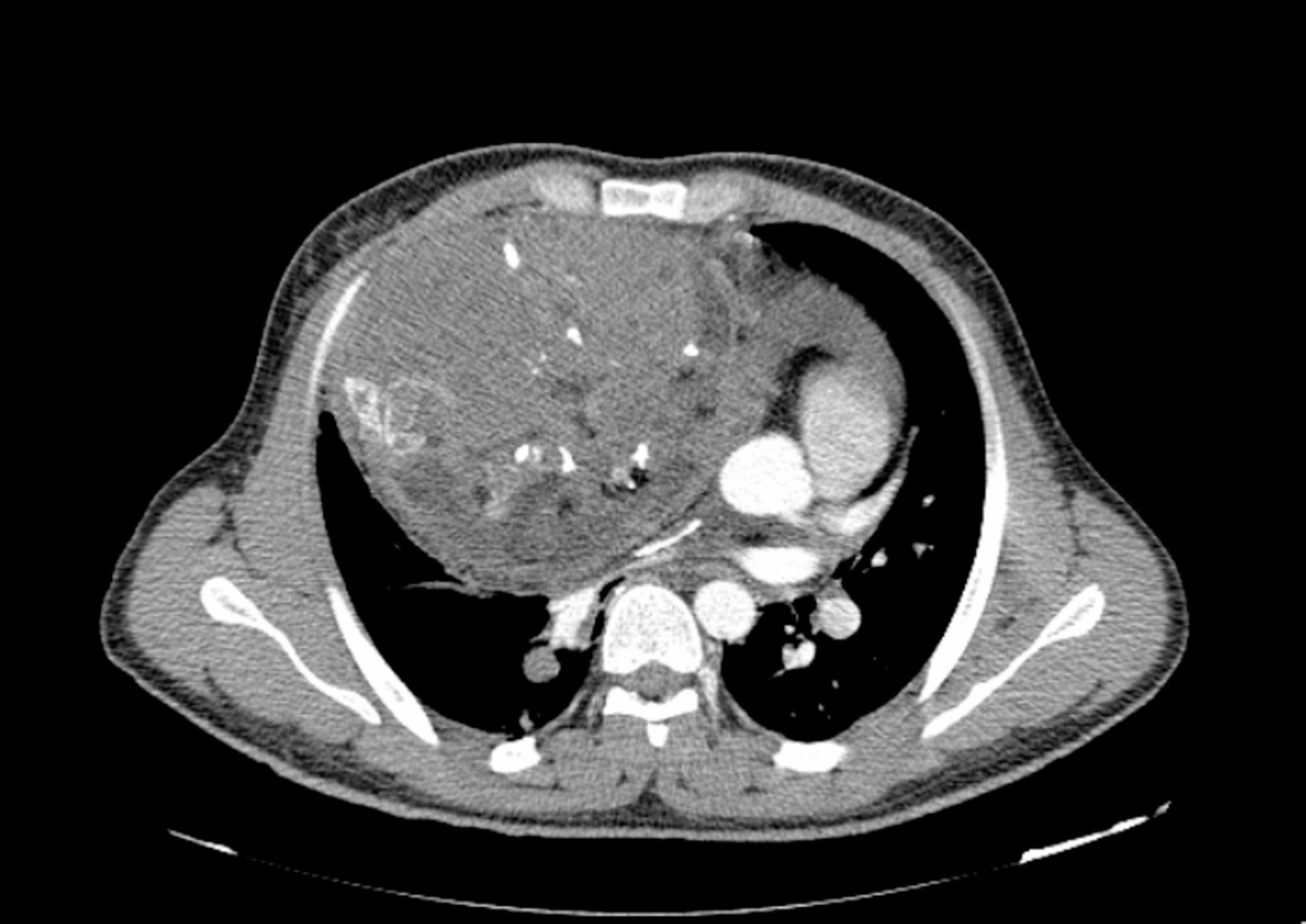
Computed tomography scan showing a large tumor in the anterior mediastinum

There, spiral computed tomography (CT) angiography of pulmonary vessels, chest x-ray, and biopsy of the mass were requested. Chest x-ray demonstrated a very large mass in the right mediastinum (Fig. 1), and the CT angiography reported a well-defined heterogeneous soft tissue density in the anterior mediastinum, anterior to the heart and great vessels, measuring about 190 × 150 × 140 mm. The mass contained fat and calcification, It caused pressure effect on the great vessels and heart, leading to heart displacement to left. Also, pressure effect on superior vena cava, right side bronchus, right pulmonary artery, and veins was seen. Besides, moderate pericardial effusion and multiple round-shaped nodules were seen in both lungs up to 14 mm in the right lower lobe, which could be metastatic lesions. Of note, prominence of both breasts in favor of gynecomastia was also detected (Fig. 2). The biopsy reported immature teratoma, at least grade II; however, the pathologist stated that the diagnosis was inconclusive, and a better evaluation had to be done after tumor excision. For unknown reasons, the patient went to another center for further evaluation.

In the second center, another HRCT was done. Pericardial tap, pericardial biopsy, and tumor biopsy were also taken; HRCT report was similar to previous HRCT report, pericardial fluid cytology was negative for malignancy, pericardial biopsy showed congestion and mesothelial cell hyperplasia with no malignant cell, and tumor biopsy was reported teratomatous tissue containing foci of immature mesenchymal and cartilaginous tissues, in favor of immature teratoma (grade I-II). Besides, another CT angiography was done, which the report was similar to the previous CT angiography. Based on the diagnosis made by tumor biopsy, the patient was referred to an oncologist.

The oncologist started chemotherapy, and the patient received four episodes of the VeIP regimen (vinblastine, ifosfamide, and cisplatin). Then, he was referred to our center with a high β- Human chorionic gonadotropin (β-hCG) and alpha-fetoprotein (α-FP) and diagnosis of immature teratoma for tumor excision. Laboratory features of the patient are demonstrated in Table 1.

**Table 1:** On admission laboratory results of a 24-year-old patient with immature teratoma of mediastinum

Test (value)	Result	Reference value	Interpretation
*White blood cell count (× 10* ^*9*^ */L)*	3.8	4.5–11	Leukopenia
*Lymphocyte percentage (%)*	45	20 − 40%	Lymphocytosis
*Neutrophil percentage (%)*	52	45–75%	Normal
*Hemoglobin (g/L)*	7.9	13.2–17.3	Anemia
*Mean corpuscular volume (fl.)*	66	80–95	Decreased
*Mean corpuscular hemoglobin (pg)*	19	27–32	Decreased
*Mean corpuscular hemoglobin concentration (g/dl)*	28	32–37	Decreased
Platelet count (x10*3/µl)	89	150–450	Thrombocytopenia
*Red cell distribution width (%)*	21	10.6–15.7	Normal
*Blood urea nitrogen (mg/dL)*	11	7–20	Normal
*Creatinine (mg/dl)*	0.9	0.5–1.4	Normal
*Lactate Dehydrogenase (IU/L)*	205	0-500	Normal
*Sodium (mEq/L)*	138	135–145	Normal
*Potassium (mEq/L)*	3.9	3.5–5.3	Normal
*Total bilirubin (mg/dl)*	1.1	0.3–1.2	Normal
*Direct bilirubin (mg/dl)*	0.3	< 0.2	Increased
*Aspartate aminotransferase (U/L)*	10	5–40	Normal
*Alanine aminotransferase (U/L)*	18	5–40	Normal
*Alkaline phosphatase (IU/L)*	217	44–147	Elevated
*Total protein (g/dl)*	6.1	6.6–7.8	Decreased
*Albumin (g/dl)*	3.8	3.8–5.1	Normal
*Globulin (g/dl)*	2.3	< 3.5	Normal
*β-Human chorionic gonadotropin (ELFA) before chemotherapy (mIU/ml)*	10.8	0–3	Elevated but declined after chemotherapy
*β-Human chorionic gonadotropin (ELFA) after chemotherapy (mIU/ml)*	3.6		
*Alpha Fetoprotein (ECLIA) before chemotherapy (mIU/ml)*	< 0.5	< 5.8	Elevated after chemotherapy
*Alpha Fetoprotein (ECLIA) after chemotherapy (IU/ml)*	13		
*SARS-CoV-2 (PCR)*	Negative	Negative	Not detected

ELFA: Equipment Leasing and Finance Association; ECLIA: electrochemiluminescence immunoassay analyzer; PCR: Polymerase chain reaction

The operation was performed under general anesthesia with a right posterolateral thoracostomy. In exploration, a huge mass with adhesion to the trachea, superior vena cava, innominate vein, esophagus, lung, pericardium, and diaphragm was observed. Also, the mass extended to the left pleura. Therefore, pneumolysis and then, partial pericardiectomy was done; and, the mass was released from its adhesive sites. Two chest tubes into the right side and one chest tube into the left side were inserted. Besides, the mass was sent for pathological evaluation.

**Fig. 3:**
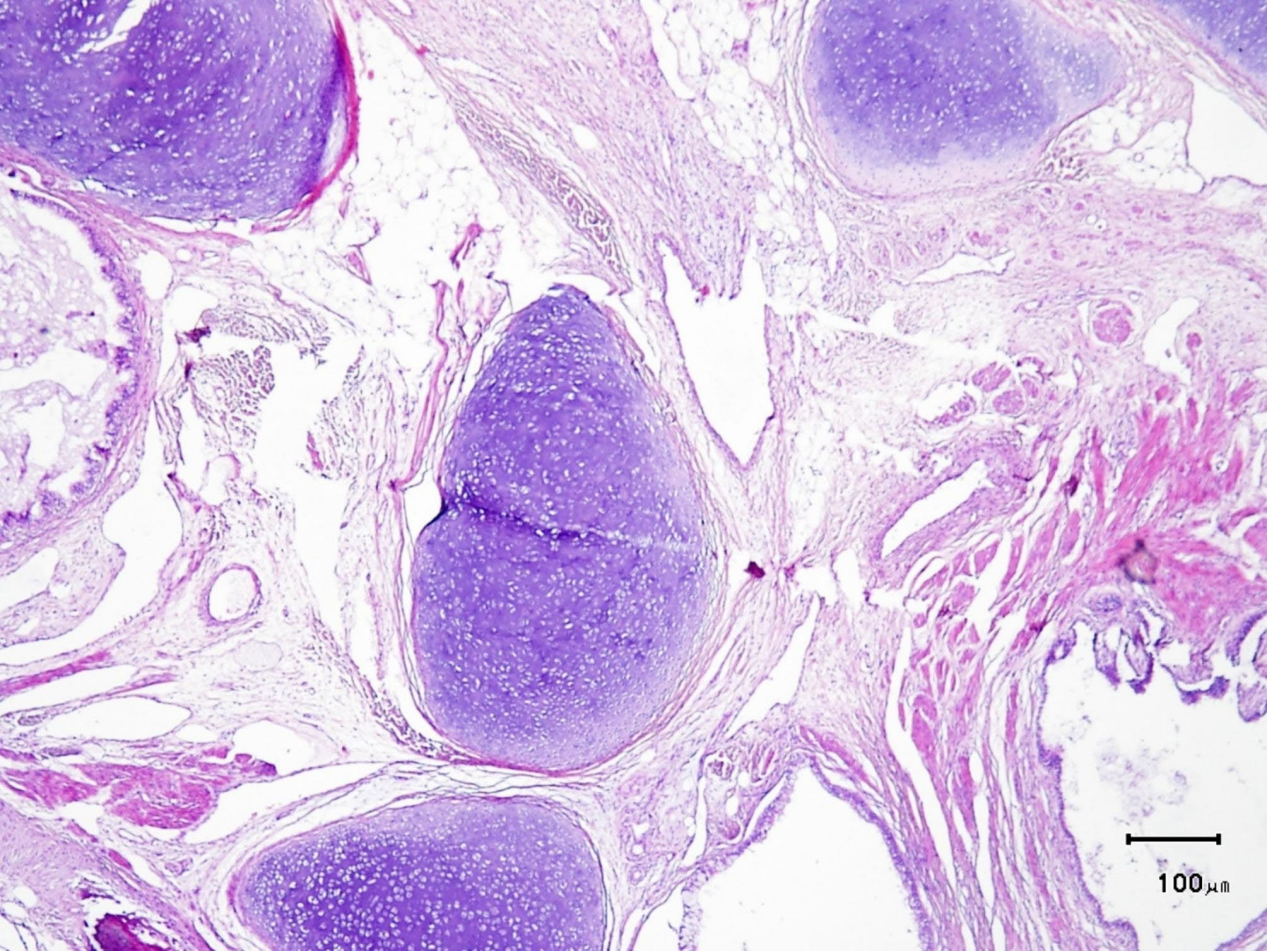
Microscopic sections showed mature cartilage, glial tissue and gastrointestinal type epithelium as well as cutaneous tissue

Grossly, the tumor size was 20 × 20 × 11 cm. In the pathological examination, serial cut sections revealed bone and cartilage formation, cystic space, some of them filled with yellow soft material and hair tuft. Also, diffuse myxoid degeneration and calcification with multiple finger-like projections covered by skin and hair were present. Finally, the definite diagnosis was reported to be an immature cystic teratoma with 10% necrosis. Microscopic sections showed mature cartilage, glial tissue, and gastrointestinal type epithelium as well as cutaneous tissue (Fig. 3).

**Fig. 4:**
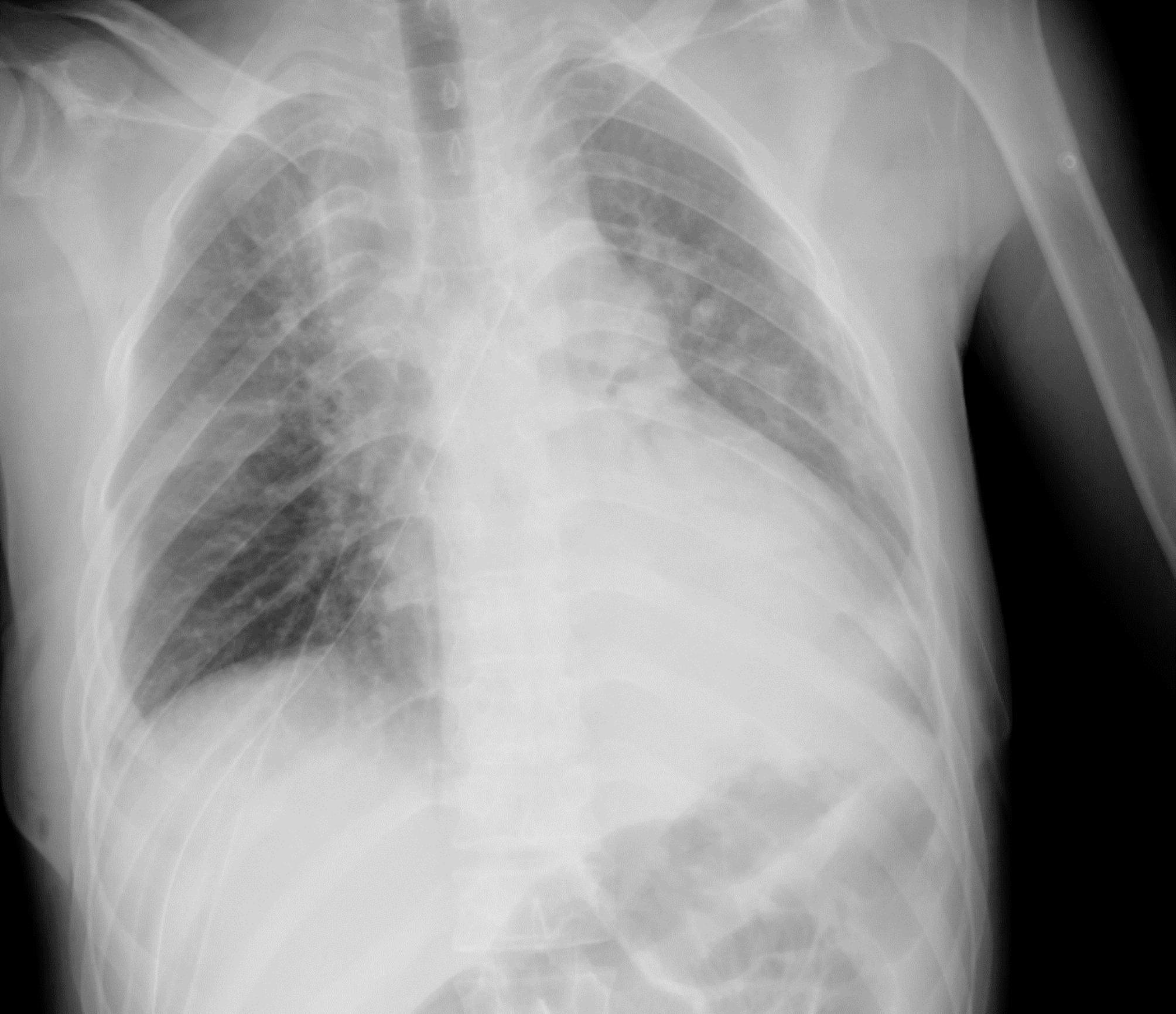
Postoperative chest x-ray after thoracotomy due to large mediastinal teratoma (4 days after the surgery)

The patient was discharged a week after the surgery in good condition (Fig. 4). Currently, he has been under our follow-up for 6 months following the operation without any signs of recurrence.

## Discussion

The anterior mediastinum is the most frequent location for extra-gonadal germ cell tumors and that teratomas are the most common form of germ cell tumors seen in the mediastinum [7]. Mediastinal teratomas are often composed of ectodermal tissues such as teeth and hair while they may also contain mesodermal and endodermal tissues. In rare cases with the presence of immature embryonic tissue in the teratoma, these tumors are regarded as immature teratoma and the management and outcome become different from a mature teratoma [2, 8]. In this report, we presented a 24-year-old patient with a giant, right-sided immature mediastinal teratoma. According to different studies, the prognosis of immature teratomas depends on various factors, including patient age and anatomical location. Mediastinal teratomas can occur in any age group [9], but the average age of presentation is around 28 years of age [10].

Because of their insidious growth, most of the mediastinal cases remain asymptomatic until they become big enough to reveal the compression effects on the nearby structures. The common symptoms of mediastinal teratomas, including substernal chest pain, dyspnea, and cough [11] followed by fever, weight loss, nausea, vena cava occlusion, and fatigue [1]. Our patient had a history of dyspnea, nausea, and chest pain for ten months.

Chest CT scan is the modality of choice for the diagnosis of mediastinal teratoma [12, 13]. It depicts the size and location of the mass, and also the extension to the surrounding structures, as well as detecting the hypervascularization of the mass [14]. Lymph nodes, germ cell tumors, and thymic and thyroid masses account for nearly all masses in the anterior mediastinum. As a result, thymoma, lymphoma, and bronchogenic cyst are among the possible differential diagnoses in our patients [15]. In CT scan, mature teratomas manifest as a unilateral huge heterogeneous mass containing fat and calcification along with areas of enhanced soft tissue or as irregular cystic tumors with thick walls and extensive hemorrhage and necrosis. (11, 15). In our case, the chest X-ray and CT scan results are compatible with the diagnosis of teratoma.

Like in our case, an elevated α-FP is not uncommon in immature mediastinal teratoma at the time of diagnosis [16]. According to some studies, unlike in a mature teratoma, the combination of cisplatin-based chemotherapy in addition to post-chemotherapy operation for complete resection has improved the prognosis [1, 16]. The effect of chemotherapy includes a decrease in tumor size, changes in tumor markers, and immature parts of the tumor. In the present case, the tumor size did not decrease after four sessions of cisplatin-based chemotherapy. Even though the final pathology report of the specimen was immature teratoma with 10% necrosis, this can be associated with the successful chemotherapy-induced necrosis in chemo-sensitive immature components and maturation differentiation in immature components of the tumor [16, 17]. It is noteworthy that our histological sample evaluation showed no components infavor of immature teratoma; however, this does not rule out the possibility that of minor immature component which responded to previous chemotherapy.

Our patient underwent surgical resection, which has been demonstrated in previous reports to be efficient in managing benign intrapulmonary and mediastinal teratomas [2, 18]. Factors supporting this decision included the large size of the mass, along with symptoms such as dyspnea and chest pain. Also, usually during operation, dense adhesions between tumors and surrounding tissues are observed, which all and all accounts for choosing the thoracotomy approach. When a lobectomy or wedge-shaped excision of the lung is required, a posterolateral thoracotomy may be chosen for a tumor in the mediastinum on one side, a large tumor, lung invasion, or pericardium. [13, 19, 20].

In conclusion, the incidence of immature mediastinal teratoma is uncommon, and due to its rarity, the diagnosis needs more profound evaluation studies such as radiological and pathological assessments. Immature teratomas are optimally treated by a combination of chemotherapy and complete resection.

## Data Availability

All data regarding this case report has been reported in the manuscript. Please contact the corresponding author in case of requiring any further information.

## Abbreviations

α-FP: alpha-fetoprotein
β-hCG: β- Human chorionic gonadotropin
CT: Computed tomography
HRCT: high-resolution computed tomography
